# Effect of Breed Types and Castration on Carcass Characteristics of Boer and Large Frame Indigenous Veld Goats of Southern Africa

**DOI:** 10.3390/ani10101884

**Published:** 2020-10-15

**Authors:** Gertruida L. van Wyk, Louwrens C. Hoffman, Phillip E. Strydom, Lorinda Frylinck

**Affiliations:** 1 Department of Animal Sciences, University of Stellenbosch, Private Bag X1, Matieland, Stellenbosch 7602, South Africa; louisa.vanwyk@virbac.com (G.L.v.W.); louwrens.hoffman@uq.edu.au (L.C.H.); pestrydom@sun.ac.za (P.E.S.); 2 Centre for Nutrition and Food Sciences, Queensland Alliance for Agriculture and Food Innovation (QAAFI), The University of Queensland, Coopers Plains, QLD 4108, Australia; 3 Animal Production, Agricultural Research Council, Private Bag X2, Irene 0062, South Africa

**Keywords:** yield, slaughter characteristics, lean meat, grazing and supplementary feeding

## Abstract

**Simple Summary:**

The purpose of this paper is to describe and compare the carcass characteristics of same-aged young wethers and bucks of Boer Goat (BG) and Indigenous Veld Goats (IVG: Cape Speckled and the Cape Lob Ear)—a collective name for the eco-types conserved by the Indigenous Veld Goat Society of South Africa. Results of this study showed that, under the same production conditions, IVG could have a similar potential for meat production. Carcass characteristics differed more between wethers and bucks than between breed types. Large frame Indigenous Veld Goat (IVG) bucks seemed particularly suited for meat production, due to higher meat yield that is leaner with lower subcutaneous and intramuscular fat, compared to the BG bucks and, in particular, to the wethers of both breed types. The wethers meat with increased subcutaneous and intramuscular fat could satisfy another consumer market segment that prefer a somewhat juicier and flavorsome meat—these aspects warrant further research. Development of the formal commercial market for goat meat would offer more diversity of species for red meat producers and especially benefit smallholder farmers who typically produce most of the goats in the world.

**Abstract:**

Weaner male Boer Goats (BG; *n* = 36; 21 bucks and 15 wethers) and large frame Indigenous Veld Goats (IVG; *n* = 41; 21 bucks and 20 wethers) were raised on hay and natural grass ad libitum and the recommended amount of commercial pelleted diet to a live weight between 30 and 35 kg. Carcass quality characteristics (live weight, carcass weights, dressing %, chilling loss and eye muscle area) were measured. The right sides of the carcasses were divided into wholesale cuts and dissected into subcutaneous fat, meat and bone. Large frame Indigenous Veld Goat (IVG) wethers were slightly lighter than the IVG bucks with no significant difference observed between BG. Wethers compared to bucks had higher dressing %, subcutaneous fat % in all primal cuts, intramuscular fat %, kidney fat % and, overall, slightly less bone %. Some breed–wether interactions were noticed: IVG wethers were slightly lighter than the IVG bucks, but the IVG bucks tended to produce higher % meat compared to other test groups. Judged on the intramuscular fat % characteristics, it seems as if wethers should produce juicier and more flavorsome meat compared to bucks.

## 1. Introduction

Goats farmed for meat production constitute the major part of the world goat population. In developed parts of the world, goats are frequently considered as specialty or exotic livestock, whereas in the developing countries, especially those in Southeast Asia and Africa, goats constitute the major source of meat production [1]. South Africa is a relatively small goat producing country contributing approximately 3% of Africa’s goat population and less than 1% of the world’s number of goats.

Little effort has been made to promote goat meat production in South Africa. Despite this, the demand for goats for traditional slaughter (i.e., slaughter of goats to mark significant occasions such as birth, coming of age, weddings, sickness, healing and death) purposes and export is rising, and in fact a shortage of goat production is experienced. Early researchers recognized the potential of the Boer Goat (BG) as a meat-producing animal [2,3], and today it is considered to be one of the most desirable goat breeds for meat production. It has gained worldwide recognition for excellent body conformation, fast growth rate and good carcass quality. Its popularity as a meat goat breed soared during the last decade due to its availability in Australia, New Zealand and later in North America and other parts of the world [4]. 

Southern Africa farming areas being a harsh environment, with challenges such as tick-borne diseases, drought and other extreme climates, require animals that are adaptable and disease-resistant. The original indigenous eco-types migrated during the fifth century AD by various means from mid Africa, endured numerous tick-borne diseases and adapted well to tropical conditions [5,6]. During the twentieth century, producers started “improving” the indigenous goats, and from there the Boer Goat was developed [7]. Unfortunately, through this development, the original indigenous eco-types nearly disappeared, and most so-called indigenous goats are actually Boer x indigenous goat crosses. Fortunately, some farmers did conserve some of the original eco-types, the Cape Speckled and the Cape Lob Ear are two of them, which were recently formally registered as Indigenous Veld Goats (IVG)—a collective name for the eco-types conserved by the Indigenous Veld Goat Society of South Africa. 

Compared to sheep and cattle, knowledge of the meat yield and quality of BG and large frame Indigenous Veld Goats (IVG, Cape Speckled and the Cape Lob Ear) of South Africa is limited due to the traditionally low commercial interest. In Africa, small ruminants (goats and, to a lesser extent, sheep) are an integral part of smallholder (subsistence) farming systems. In these operations, goats and sheep make a significant contribution to the total farm income, the stability of farming systems and human nutrition [8]. These smallholder farming systems mainly perform under communal situations, which refer to where large areas of state rangeland (veld) are used communally by farmers for grazing domestic livestock and harvesting natural products [9]. Nonetheless, there are commercial opportunities within the goat industry in South Africa that could be developed to increase the income of rural populations [10]. In fact, interest has also grown for the potential of rounding off of goats in feedlots [11,12,13].

The acceptability of a carcass lies in its perceived economic value, which includes the potential meat yield of the carcass [14]. Although live animal and carcass attributes are principally concerned with the quantity of saleable meat that can be obtained from the carcass, they also have significant implications on the technological value of the carcass (i.e., the morphology of some specific muscles and cuts). These attributes influence the biochemical and physiological processes in meat during slaughter and chilling, and hence the resultant quality of the meat [15]. Therefore, early identification of animal characteristics that affect meat quality is beneficial for the production of meat of acceptable quality. Traits such as the sex, age, weight and conformation of the live animal and carcass as well as the fat distribution in the carcass are therefore of importance in producing goat meat of acceptable quality. The proportion of high-value cuts is also an important indication of the overall value of the carcass [16,17], yet little data exist on these carcass attributes for both the BG and IVG. The purpose of this paper is to describe and compare the carcass characteristics of same-aged young wethers and bucks of BG and IVG (Cape Speckled and the Cape Lob Ear).

## 2. Materials and Methods 

### 2.1. Animals and Experimental Design

**Figure 1:**
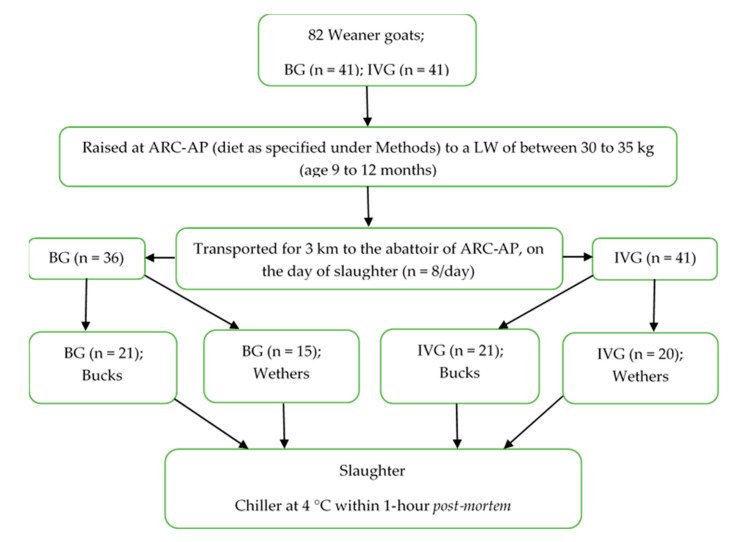
Experimental design to determine yield of Boer Goats (BG) and large frame Indigenous Veld Goats (IVG), bucks and wethers slaughtered at a pre-determined weight (30 to 35 kg); ARC-AP—Agricultural Research Council—Animal Production, Irene, South Africa.

This research was approved by the Agricultural Research Council—Animal Production (ARC-AP) Ethics Committee (ref no. APIEC16/021). Weaner Boer Goats (BG; *n* = 41; 21 bucks and 20 wethers) and large frame Indigenous Veld Goats (IVG; *n* = 41; 21 bucks and 20 wethers) were purchased from commercial breeders at three months of age (17 kg on average for IVG and 20 kg on average for BG). The commercial breeder when bought in had already castrated the male animals on farm. The animals were reared at the at the Small Stock Section of the Agricultural Research Council—Animal Production (ARC-AP) facility situated in Irene, in the Gauteng province of South Africa. During the rearing phase the goats were randomly placed (breed and sex mixed) in two similar large grazing camps (~1500 m^2^) with similar natural grass available during the summer rainfall areas in South Africa, with enough space to move and graze without affecting each other. From time to time they were moved to other camps, when the grass seem to be withered and to lessen the chance of worm infestation. The aim was to simulate a small farm situation that is typical in the grassland areas. The natural grass diet was supplemented with hay ad libitum and an average of 250 g commercial “Ram, lamb and ewe—13” pellets (protein 130 g/kg, fat 25–70 g/kg, fiber 150 g/kg, moisture 120 g/kg, calcium 15 g/kg, phosphorus 3 g/kg, urea 10 g/kg; Meadow Feeds, Lanseria Corporate Estate, Malibongwe Drive, Lanseria, Gauteng, South Africa) per day per animal. Pellets were spread evenly along 20 m long narrow feeding troughs, giving all goats in the camps an equal opportunity to feed. Water was provided ad libitum. The duration spent under these farming conditions was on average 6 to 8 months to a live weight (LW) of between 30 and 35 kg. After weighing (LW), the goats were transported for 3 km to the abattoir of the ARC-AP on the day of slaughter. The experimental design is presented in Figure 1. 

### 2.2. Slaughter and Sampling Procedures

**Figure 2:**
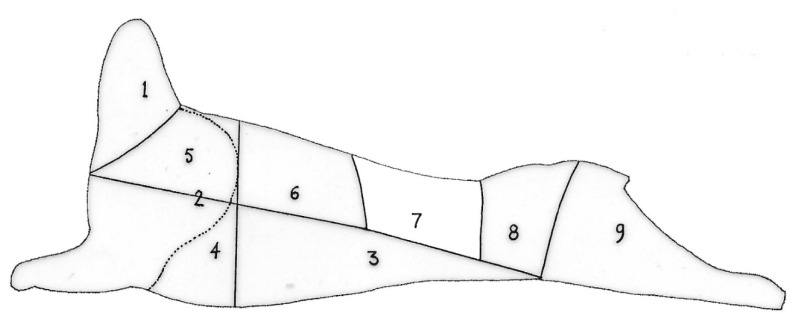
Dissection diagram representing goat carcass composition. 1—neck (cranial end); 2—thick rib; 3—flank (abdominal muscles); 4—shoulder; 5—breast; 6—lower rib; 7—loin; 8—chump; 9—leg and shin (caudal end) [20].

A maximum of eight goats per day representing all experimental groups were slaughtered. After a lairage period of 2 h, the goats were rendered unconscious by electrical stunning (5 s at 200 volts, 0.5 A), exsanguinated and carcasses suspended by both Achilles heels and allowed to bleed out for 5 min [18]. The head was removed after evisceration by further cutting the neck and was severed from the spinal column at the occipito–atlantal joint. The trotters were removed by severing the joint between the metacarpus and the radius/ulna in the forelimbs and severing the joint between the metatarsus and the tibia/fibula in the hind limbs. The red offal was plucked from the abdominal cavity during evisceration. The red offal consisting of the heart, liver, lungs and spleen was not part of this study and was discarded. The warm carcass weight (1 h post-mortem) was recorded before the carcasses were suspended from both hind legs in the chiller. All carcasses were placed in the chiller at 4 °C within 60 min post-mortem. The carcasses were classified according to the South African Carcass Classification for Small Stock (SACCSS) according to age, fat cover and conformation [19]. Cold carcass weights (24 h post-mortem) were measured. The kidney fat and kidneys were plucked from the abdominal cavity, and then the chilled carcasses were sectioned down the vertebral column by band saw after removing the tail. Both the kidneys and kidney fat were weighed for each carcass. The left side was subdivided into ten South African retail cuts, i.e., neck, shoulder and shank, breast, rib, loin, chump, leg, shin and tail following the method of Strydom et al. [20] as indicated in Figure 2.

The cut weights were recorded to the nearest gram, and each cut was deboned and subcutaneous fat (SCF) removed. Weights of meat (including muscle, intermuscular and intramuscular fat), bone (including large sinews and cartilage) and SCF were recorded to calculate the physical composition of each cut and of the carcass side [20]. Eye muscle areas were measured in mm^2^ from traced images of the longissimus thoracis (LT) muscle on the surface of the cut made at the first lumbar vertebrae (L1) using a Video Image Analyser equipped with a XC30 Colour Camera (Olympus Soft Imaging Systems Gmbh, Münster, Germany), and cellSens Life Science Imaging Software (Olympus Corporation, Tokyo, Japan) after calibrating the X and Y axes. Moisture, protein, fat (representing chemical determined intramuscular fat; IMF) and ash percentages of lean loin meat were analyzed using the procedures of the Association of Official’s Analytical Chemist [21] at the Analytical Laboratories, ARC-AP, Irene, South Africa.

Dressing percentages (DP) and chilling losses of goat carcasses were calculated as follows:

- $\mathrm{DP}(\%)=\frac{\mathrm{CCW}}{\mathrm{LW}}\times 100$
- $\mathrm{Chilling}\;\mathrm{loss}\;(\%)=\frac{\mathrm{HCW}-\mathrm{CCW}}{\mathrm{HCW}}\times 100$

where CCW = cold carcass weight; LW = live weight at slaughter; HCW = hot carcass weight.

### 2.3. Statistical Analysis

The data were subjected to analysis of variance [22] using a two-way ANOVA to test the effect of the two goat breeds (B) (BG and IVG), two sex-types (S) (buck and wethers) and interactions as factors on live weight, carcass weight and other carcass characteristics [23]. Slaughter date and age (presence of number of teeth) as random effects had no effect on the outcome of the results (*p* > 0.05) and thus will not be mentioned further. Five BG wethers died during the study due to wilted grass, anemia and coccidiosis, causing an unbalanced dataset (see Figure 1—experimental design).

Prior to analyses, a Shapiro–Wilk test for normality was performed on the data [24], and where applicable, outliers were classified when the standardized residual for an observation deviated with more than three SDs from the model value. Few outliers for specific parameters were removed as specified in tables in brackets under means. Whole animal data were not removed. Statistical significance (Fisher’s t-test, least significant difference) was calculated at a 5% level to compare means. *p* < 0.05 was considered statistically significant, although in some instances data with a *p* ≤ 0.1 (10% level) was considered as a trend worth discussing.

## 3. Results and Discussion

When evaluating the commercial cuts in this study, % of cuts per carcass weight of the neck, thick rib, loin and leg cuts showed differences (*p* < 0.05), in addition for shoulder a tendency (*p* ≤ 0.10) between breed x sex interactions (Table 1). On the other hand, the neck and chump differed (*p* < 0.05) between sexes and the neck, flank, breast and tail differed (*p* < 0.05) between breeds. 

### 3.1. Carcass Characteristics

Only a few of the parameters measured showed a breed x sex interaction (Table 1), and where applicable these will be discussed. Where there were no interactions, the main effects are discussed further. Generally, the evaluation of carcass characteristics and yield carcasses of the two breed types (BG vs. IVG) from wether and buck male goats showed more differences between the sexes (bucks vs. wethers) than between the breeds (Table 1). 

There was a tendency to differ (*p* = 0.070) between breed and sex for the live weight at slaughter of the goats. The average LW of IVG wethers was lighter than that of the IVG bucks, although neither of the two IVG sexes differed from the BG bucks and wethers. On the other hand, there was no interaction (*p* = 0.130) between the breed x sex for the warm carcass weight. The average warm carcass weight of the BG wethers was higher than that of the BG bucks and tended (*p* = 0.063) to differ between the two sexes; whilst that of the IVG did not differ between the two sexes. This ranking order was also continued with respect to the cold carcass weights. The mean values of cold carcass weight for BG (bucks = 14.8 kg; wethers = 15.8 kg) were in line with the mean values previously recorded for BG fed on different energy diets (low energy diets = 15.28 kg; high energy diets = 17.05 kg) [16]. 

Boer Goats (BG, both sexes) presented significant (*p* = 0.011) higher chilling losses (≥3.5%) compared to that of the IVG wethers (3.0%) (Table 1), but similar to IVG bucks (3.3%). Chilling losses in goat carcasses are normally in the range of 2.3% to 3.0%, and the loss tend to be higher compared to sheep carcasses at comparable ages and sexes [25], a phenomenon attributed to the absence of thinner subcutaneous fat cover (SCF) found in goats. The goat carcasses were all classified as being fat codes between −1 and 1 according to the South African Carcass Classification for Small Stock [19], and the specific depth of the SCF was not measurable in the present investigation. Goats are late maturing compared to sheep and grow at a slower rate; thus, fat is only deposited as they progress in chronological age and/or weight [12,25,26,27]. Goat meat is generally considered a lean meat that is an ideal protein source for health-conscious groups that try to limit their fat intake. Nonetheless, IVG wethers had the lowest chilling loss (Table 1) and the highest proportions of SCF in all of the commercial cuts (Table 2), a finding that supports the argument that higher levels of SCF reduce chilling losses [28,29,30]. High chilling losses are undesirable as they reduce the weight and the economic value of the carcasses as seen between the BG bucks and IVG wethers. 

The mean dressing percentage (DP) varied between 41.9% and 44.9%, which generally agrees with the values reported for various goat breeds worldwide [31,32]. Both the BG and IVG bucks had 3% to 5% lower (*p* < 0.001) DP compared to wethers, while there was no breed effect (Table 1). Dressing percentage is both a yield and financial value-determining factor [33] and is affected by factors such as age, weight, level of nutrition, the degree of gut fill at slaughter, head and skin weight, fatness and dressing procedures [32,34,35]. Castration slows down an animal’s growth by increasing the rate of deposition of adipose tissue (fat) to the detriment of the muscular tissue (meat) on the carcass at slaughter [34]. This phenomenon also explains the higher percentage of kidney fat in wethers compared to that of the bucks in the current study (Table 1). In addition, it is likely one of the main reasons for differences in DP between the two sexes. Factors such as gut fill, head and skin weight were not measured in the current study and should be considered in future studies to define the impact on DP between BG and IVG. Dressing percentages of goats are usually between 35% and 53% [36], although DP toward the higher end of the range of between 42% and 45% have been reported for Boer and undefined South African indigenous goats [37], which is in agreement with the current study (Table 1). 

There was a trend (*p* = 0.053) for interactions between the breeds and sexes for the eye muscle area (EMA). Boer Goat (BG) wethers had the largest EMA whilst the IVG wethers had the smallest. The bucks of both breeds (BG and IVG) had intermediate EMAs that did not differ from each other (Table 1). An interesting observation is that the IVG wethers had the smallest EMA but presented the group with heavier loins. This could be caused by longer loins and/or more subcutaneous fat being laid down in the loin region (Table 2). Further research would need to be conducted to compare breeds and sexes from different eco-types and determine whether longer loins could be more than a casual observation to indicate that the IVG wether goats had longer carcasses. A phenomenon known to be associated with time of castration, pubertal development and the change caused by in cycling androgens and estrogen cycling is reported [38]. The animals in this study had all been castrated by the age of three months when they entered the trial, although the specific age of castration is not known. This phenomenon should be studied further as the length of the carcass will have an influence on the weight of high-value cuts available for sale.

### 3.2. Commercial Cuts and Proportions of Tissue Composition

The changes related to goats’ body conformation are associated with the onset of puberty. With the onset of puberty, animals start to develop secondary sexual characteristics, which adapt the musculature for survival and reproduction [39]. Therefore, it can be expected that sexually mature bucks will have a more developed neck and thorax, while does will exhibit a greater rump region to aid with birth [39]. Castrated goats are found to still exhibit body shape changes associated with puberty in intact males [27], although to a lesser extent, while generally exhibiting a higher degree of fatness [40].

The IVG bucks recorded the highest % yields for the neck than all other groups, but similar % yields were recorded for the other cuts such as the thick rib and shoulder than that of BG bucks and wethers. On the other hand, the IVG wethers differed in % yields where the thick rib and shoulder had the lowest % yield and the loin and leg had the highest % yield compared to the IVG buck and BG bucks and wethers (Table 1). The similarities between BG bucks and wethers can be explained by the fact that the wethers and bucks used in this study were not yet at mature adult weights when slaughtered. It would be interesting to see at what physiological age and/or weight these goat breeds reach their mature status and whether the rules of Berg and Walters apply [36]. For both the breast and the flank cuts, the BG recorded higher yields than the IVG. Boer Goats (BG) had a significant higher flank % (>6.8%), breast % (>12.1%) and tail % (0.62%) compared to IVG (<6.5%, <11.7% and 0.56 %, respectively) of carcass weight. For the averages between the values shown in Table 1, a significant breed difference was observed for the neck, with IVG (14.5%) presenting higher yields compared to BG (13.4%). When evaluating differences between the sexes, both chump % and leg % of carcass weight differed significantly with higher yields observed for wethers (7.3% and 18.9%, respectively) to that of bucks, whereas bucks presented higher yield in terms of % neck of the carcass (14.5%). No significant differences were observed for the % shin in terms of an interaction between the breeds and the sexes, nor for the main effects evaluated. 

Wethers recorded higher proportional yields for kidney fat irrespective of breed; however, IVG had a tendency to yield higher percentages of kidney fat compared to BG. Generally, wethers tend to be fatter than bucks [41], although, unlike lamb, goats have relatively lower levels of subcutaneous and intramuscular fat [16,42,43]. 

The proportions of tissue composition dissected (bone, subcutaneous fat and meat as % of each primal cut) and comparison of yield means of primal cuts (kg) from BG and IVG, wethers and bucks are presented in Table 2.

Typically, goat carcasses have more than 60% dissectible lean meat and 5% to 14% dissectible fat [37]. Subcutaneous fat is poorly developed in goats, and fat accretion occurs at a later stage in the growth process compared to other livestock species [25]. This was also reflected in this study when comparing an average of 63% lean, 22% bone, 10% intermuscular fat and 5% subcutaneous fat reported for whole carcasses [44]. However, the current study presented higher SCF % (5.9% to 10.5%) compared to reported values of 2.7% to 5% [44]. An explanation could be that the animals used in the study of Simela [44] were slaughtered at a weight of at least 25 kg (6 to 10 months) vs. 30 to 35 kg (9 to 12 months), supporting that pre-slaughter conditioning (to slaughter at a later stage in the growth process) improved fat/bone indices. 

Boer goat (BG) wethers and bucks showed no differences in fat and meat, while, for IVG, wethers recorded higher fat and lower meat proportions than bucks (Table 3). Low carcass fat is one of the main attractions to goat meat production. However, the low and rather variable subcutaneous fat cover is a particular cause for concern in commercial goat meat production since it is often well below the levels considered necessary for effective carcass chilling, without the risk of cold shortening [45,46]. The lean carcasses, coupled with the faster growth of the bucks, are the basis for the drive to produce young bucks in preference to wethers. However, at sexual maturity and beyond, meat from bucks is believed to have an unacceptably strong odor caused by androgens and branched-chain fatty acids [47], which leads to the downgrading of their carcasses.

The general trend in commercial goat production is to use cuts similar to that in lamb [48]. The effectiveness of this in marketing goat meat is debatable since the two species differ in distribution of joints within the carcass as well as the dissectible tissues within the joints [3]. Previous research shows that the preference for the cuts varies with cultural backgrounds. Whereas, in most of the western world, cuts from the hind limb and the dorsal region are of prime value and the breast region is of lower value, whilst a high preference for the breasts has been shown in some studies conducted in Africa and Asia [48,49]. An understanding of the market needs within each country, taking into consideration the different eco-types (genotypes) available, is therefore essential for the development of a market for goat meat. 

When evaluating the primal cuts and the weight that each cut contributed to the total carcass (Table 2), the neck, thick rib and shoulder as primal cuts had a significant weight interaction between the breeds and sexes. Large frame Indigenous Veld Goat (IVG) bucks had heavier necks (1.19 kg) than IVG wethers as well as BG bucks and wethers. For both thick rib and shoulder, BG wethers were heavier (0.56 kg and 1.02 kg, respectively) than BG bucks as well as IVG bucks and wethers. All primal cuts having SCF had significant interactions (breed x sex) except the flank. IVG bucks always seemed to be trending lower, with the highest percentages measured in IVG wethers. On the other hand, the opposite observation was made for muscle % where IVG bucks had significant higher percentages for the neck (73.9%) and shoulder (76.7%), with a tendency observed in the breast (63.4%), compared to IVG wethers and BG (wethers and bucks). In addition, BG wethers presented higher % muscle for the leg (79.0%) and a tendency to be higher for the chump (72.0%). No significant interactions for % bone was observed, apart from a tendency for the leg and chump, with higher percentages observed for BG bucks followed by IVG bucks, IVG wethers and BG wethers. The proportion of bones in most joints could be explained by the early maturing nature of bone tissue [50]. Bone matures early in the lifetime, such that its turnover rate is slower than that of fat and muscles later in life [51].

When considering the main effects, wethers in general had higher percentages of SCF (neck, flank, shoulder, breast, loin and chump) compared to bucks; however, bucks had higher percentages of bone (thick rib, loin, chump and leg) and muscle (flank, shoulder and breast). Large frame Indigenous Veld Goats (IVG) significantly had higher % bone for the shoulder (>19.5%) and shin (>40.8%) with a tendency to have higher % muscle in the neck and breast compared to BG. No significant breed differences were observed for SCF % in all the primal cuts. Within the carcasses, overall, the leg and shoulder seem to be the most ideal high-value cuts in terms of saleable meat yield due to their exceptional lean and low fat levels, although the possibility exists that the quality (particularly tenderness) of these cuts might not be ideal.

### 3.3. Proximate Composition of Loins

There were no interactions for any of the proximate chemical composition between breed and sex (Table 3) after the removal of the SCF. There were sex effects for moisture, protein, fat and ash percentages. In addition, significant breed effects were observed for fat and ash percentages, whereas no significance was observed in terms of moisture and protein. Values noted in this investigation correspond to that reported elsewhere [52]. 

Both BG and IVG bucks had higher % moisture, whilst BG and IVG wethers had higher % fat. The IVG wethers demonstrated higher values for kidney fat (Table 1) in combination with more subcutaneous fat in the various commercial cuts (Table 2), and they can be associated with a higher order of development of various fat depots [39]. Goats deposit more visceral fat and less subcutaneous, inter- and intramuscular fat compared to sheep and cattle [25]. Several studies have compared the chemical composition of sheep and goats at the same slaughter weight, age or under similar feeding management and have found that goat meat is characterized by lower intramuscular fat and higher moisture content [53,54,55,56]. Even though significant differences (*p* = 0.001) for ash % were detected between the breeds and between sexes, the % were still low (0.9% to 1.1%). Although there is documentation on chemical composition and meat quality of sheep and goat meat [16,56,57], the results from the current study highlight differences between Indigenous goat eco-types and breeds in South Africa. This could be an area for further exploration as has been done with different sheep breeds.

## 4. Conclusions

Although the Boer Goat (BG) is the most popular goat breed across the world for meat production, the results of this study showed that, under the same production conditions, Indigenous Veld Goat (IVG) could have a similar potential for goat meat production. More significant differences in carcass characteristics were observed between the wethers and bucks rather than between breed types. Large frame IVG bucks seemed particularly suited for higher meat yield that is leaner with lower subcutaneous and intramuscular fat (SCF and IMF), compared to the BG bucks and, in particular, the wethers of both breed types. The latter tend to accumulate more SCF and IMF. On the other hand, wethers produce a meat product (chevon) with increased SCF and IMF contents that could satisfy another consumer market segment that prefer a somewhat juicier and flavorsome carcass—these aspects warrant further research. Development of the formal commercial market for goat meat would offer more diversity of species for red meat producers and especially benefit smallholder farmers who typically produce most of the goats in the world. 

## Figures and Tables

**Table 1:** Least-square means and standard error (SE) of means for carcass characteristics of Boer and large frame Indigenous Veld buck and wether goats (number of outliers removed indicated in brackets).

	Breed						
Carcass Characteristics	BG		IVG		Significance (*p*-Values)		
	Bucks *n* = 21	Wethers *n* = 15	Bucks *n* = 21	Wethers *n* = 20	Breed	Sex	Breed × Sex
Live weight (kg)	35.5 ^xy^ ± 3.26 (1)	35.7 ^xy^ ± 2.91 (1)	36.4 ^y^ ± 2.09	34.3 ^x^ ± 2.38 (1)	0.748	0.114	0.070
Warm carcass weight (kg)	15.4 ± 1.48	16.4 ± 2.08	15.8 ± 0.73	15.9 ± 1.20	0.918	0.063	0.130
Cold carcass weight (kg)	14.8± 0.48 (2)	15.8 ± 1.40	15.2 ± 0.72 (1)	15.4 ± 1.19	0.774	0.055	0.164
Chilling loss (%)	3.5 ± 0.52 (2)	3.5 ± 0.57	3.3 ± 0.50 (1)	3.0 ± 0.56	0.011	0.221	0.125
Dressing %	41.9 ± 2.69 (1)	44.2 ± 1.12 (1)	41.9 ± 2.49	44.9 ± 2.06 (1)	0.347	<0.001	0.580
Eye muscle area (mm^2^)	1043 ^xy^ ± 265	1184 ^y^ ± 269	1049 ^xy^ ± 242	964 ^x^ ± 194	0.101	0.732	0.053
Commercial cuts (% of carcass weight):							
Neck (%)	13.5 ^a^ ± 1.4	13.3 ^a^ ± 1.7	15.6 ^b^ ± 1.8	13.4 ^a^ ± 0.9	0.001	0.014	0.002
Thick rib (%)	6.5 ^bc^ ± 0.9	7.2 ^b^ ± 1.0	7.1 ^ab^ ± 1.2	6.4 ^a^ ± 0.7	0.824	0.859	0.005
Flank (%)	6.9 ± 0.8	6.8 ± 1.2	6.1 ± 6.1	6.5 ± 0.2	0.015	0.475	0.363
Shoulder (%)	12.9 ^y^ ± 0.6 (1)	13.1 ^y^ ± 0.7	12.9 ^y^ ± 0.9 (1)	12.6 ^x^ ± 0.8	0.123	0.816	0.096
Breast (%)	12.1 ± 0.8	12.3 ± 0.7 (1)	11.8 ± 0.7	11.7 ± 0.6 (1)	0.005	0.715	0.403
Loin (%)	12.7 ^ab^ ± 12 (1)	12.0 ^a^ ± 1.3	12.2 ^b^ ± 1.0	13.1 ^b^ ± 1.2 (1)	0.359	0.629	0.003
Chump (%)	7.0 ± 0.6	7.2 ± 0.4	6.8 ± 0.4	7.4 ± 0.6	0.183	<0.001	0.230
Leg (%)	18.4 ^a^ ± 1.3	18.3 ^a^ ± 1.4	18.1 ^a^ ± 1.2	19.3 ^b^ ± 0.7	0.231	0.022	0.007
Shin (%)	9.7 ± 0.7 (1)	9.4 ± 1.2 (1)	9.2 ± 0.8	9.2 ± 0.8	0.115	0.585	0.376
Tail (%)	0.6 ^a^ ± 0.1	0.6 ^a^ ± 0.1	0.5 ^b^ ± 0.1	0.6 ^a^ ± 0.1	0.048	0.088	0.049
Additional (% of kidney and kidney fat together)							
Kidney %	23.4 ^xy^ ± 4.4	19.4 ^y^ ± 4.4 (2)	22.7 ^xy^ ± 6.9	16.7 ^x^ ± 5.7 (1)	0.576	0.415	0.076
Kidney Fat %	76.6 ± 4.4 (1)	80.6 ± 4.4 (2)	77.3 ± 6.9	83.3 ± 5.7 (1)	0.062	<0.001	0.745

^a,b^ Means within the same row with different letters differ (*p* < 0.05), and ^x,y^ means differed with *p* ≤ 0.10 and was considered to be a trend, both reflecting the breed x sex level; Significant *p*-values are presented in bold; BG—Boer Goat; IVG—large frame Indigenous Veld Goat.

**Table 2:** Least-square means and standard error (SE) for proportions of tissue composition dissected (bone, subcutaneous fat and muscle as % of each primal cut) and comparison of yield means of primal cuts (kg) of Boer and large frame Indigenous Veld buck and wether goats (number of outliers removed indicated in brackets).

		Breed						
		BG		IVG		Significance (*p*-Values)		
Prime Cut Composition		Bucks	Wethers	Bucks	Wethers	Breed	Sex	Breed × Sex
Total meat (%)		69.4 ^b^ ± 2.6	69.5 ^b^ ± 1.8	71.0 ^c^ ± 3.3	67.4 ^a^ ± 1.5	0.790	0.555	0.015
Total bone (%)		22.7 ± 2.0	21.5 ± 1.7	23.1 ± 1.4	22.2 ± 1.4	0.240	0.008	0.690
Total subcutaneous fat (%)		7.9 ^b^ ± 1.6	9.1 ^b^ ± 1.8	5.9 ^a^ ± 2.3	10.5 ^c^ ± 1.7	0.639	<0.0001	0.003
Primal cuts and primal cut tissue composition:								
Neck	Total (kg)	1.0 ^a^ ± 0.16	1.0 ^a^ ± 0.18	1.2 ^b^ ± 0.13	1.0 ^a^ ± 0.09	0.006	0.014	0.001
	Bone (%)	18.5 ± 3.0	17.6 ± 3.2	18.2 ± 2.8	18.7 ± 2.6	0.646	0.841	0.310
	Subcutaneous fat (%)	13.0 ^b^ ± 3.9 (2)	15.3 ^b^ ± 2.8	7.9 ^a^ ± 3.6	15.3 ^b^ ± 3.7 (1)	0.004	<0.0001	0.003
	Muscle (%)	68.4 ^a^ ± 5.9 (2)	67.1 ^a^ ± 5.4 (1)	73.9 ^b^ ± 4.1	66.0 ^a^ ± 4.40	0.065	<0.0001	0.007
Thick rib	Total (kg)	0.5 ^a^ ± 0.11	0.6 ^b^ ± 0.12	0.5 ^ab^ ± 0.10	0.5 ^a^ ± 0.06	0.868	0.841	0.006
	Bone (%)	30.1 ± 4.6	28.4 ± 3.3	31.4 ± 3.7	29.3 ± 3.1	0.258	0.027	0.837
	Subcutaneous fat (%)	6.8 ^b^ ± 1.5	7.0 ^b^ ± 2.1	5.1 ^a^ ± 0.5	8.5 ^c^ ± 2.1	0.767	<0.0001	0.001
	Muscle (%)	63.1 ± 5.2 (1)	64.6 ± 4.0 (1)	63.5 ± 4.0	62.2 ± 3.6	0.383	0.980	0.144
Flank	Total (kg)	0.5 ± 0.09	0.5 ± 0.12	0.5 ± 0.08	0.5 ±0.09	0.075	0.317	0.932
	Subcutaneous fat (%)	16.5 ± 5.9	20.7 ± 7.7 (2)	15.6 ± 5.9	23.6 ± 6.9 (1)	0.414	<0.0001	0.216
	Muscle (%)	83.3 ± 6.0	79.1 ± 7.6	84.3 ± 5.9	76.4 ± 6.9	0.473	<0.0001	0.238
Shoulder	Total (kg)	1.0 ^b^ ± 0.10 (1)	1.0 ^a^ ± 0.10	1.0 ^ab^ ± 0.10 (1)	1.0 ^b^ ± 0.10	0.652	0.535	0.028
	Bone (%)	18.6 ± 1.4 (1)	18.5 ± 1.8	19.5 ± 1.7 (1)	19.5 ± 1.9 (1)	0.020	0.807	0.951
	Subcutaneous fat (%)	5.3 ^ab^ ± 3.0 (1)	5.5 ^ab^ ± 2.7	3.8 ^a^ ± 1.5 (1)	7.3 ^b^ ± 3.5	0.886	0.004	0.001
	Muscle (%)	76.1 ^b^ ± 3.1 (1)	76.0 ^b^ ± 3.9	76.7 ^b^ ± 1.8 (1)	73.3 ^a^ ± 3.1	0.147	0.010	0.020
Breast	Total (kg)	0.9 ^x^ ± 0.10	1.0 ^y^ ± 0.11 (1)	0.9 ^xy^ ± 0.07 (1)	0.9 ^x^ ± 0.08	0.183	0.319	0.080
	Bone (%)	28.8 ± 2.9	27.8 ± 3.5 (1)	28.6 ± 1.6 (1)	27.3 ± 2.9	0.501	0.070	0.808
	Subcutaneous fat (%)	11.0 ^b^ ± 3.5	12.2 ^b^ ± 3.7 (1)	8.1 ^a^ ± 3.6 (1)	12.9 ^b^ ± 3.6	0.224	<0.0001	0.032
	Muscle (%)	60.3 ^x^ ± 4.0	60.1 ^x^ ± 3.6 (1)	63.4 ^y^ ± 3.2 (1)	59.8 ^x^ ± 3.7	0.093	0.019	0.053
Loin	Total (kg)	0.9 ± 0.16 (1)	0.9 ± 0.12	0.9 ± 0.09	1.0 ± 0.14 (1)	0.380	0.418	0.185
	Bone (%)	25.3 ± 5.2	24.5 ± 4.4	27.4 ± 3.7	23.7 ± 0.79	0.532	0.014	0.146
	Subcutaneous fat (%)	6.7 ^bc^ ± 3.3 (1)	8.7 ^b^ ± 2.9	4.7 ^a^± 3.9 (3)	11.4 ^c^ ± 4.0	0.624	<0.0001	0.005
	Muscle (%)	67.9 ± 4.9 (1)	66.9 ± 6.1	68.0 ± 4.5	64.9 ± 4.7	0.390	0.066	0.386
Chump	Total (kg)	0.5 ± 0.07	0.6 ± 0.08	0.5 ± 0.04	0.6 ± 0.05	0.720	0.004	0.733
	Bone (%)	24.2 ^y^ ± 4.6	20.3 ^x^ ± 3.5	22.6 ^xy^ ± 3.6	22.6 ^xy^ ± 3.6	0.840	0.027	0.058
	Subcutaneous fat (%)	7.4 ^ab^ ± 2.2	7.7 ^ab^ ± 2.7	6.6 ^a^ ± 2.2	9.2 ^b^ ± 2.6	0.512	0.007	0.040
	Muscle (%)	68.5 ^x^ ± 4.5	72.0 ^y^ ± 4.8	70.8 ^xy^ ± 3.3	68.7 ^x^ ± 4.6	0.849	0.655	0.055
Leg	Total (kg)	1.4 ± 0.12	1.4 ± 0.12	1.4 ± 0.11	1.5 ± 0.11	0.132	0.012	0.554
	Bone (%)	17.6 ^y^ ± 1.9	15.9 ^x^ ± 2.3	17.6 ^y^ ± 1.8	17.4 ^y^ ± 1.5	0.158	0.046	0.085
	Subcutaneous fat (%)	5.3 ^a^ ± 1.6	5.1 ^a^ ± 2.2	4.2 ^a^ ± 1.5	7.4 ^b^ ± 1.9	0.190	<0.0001	<0.0001
	Muscle (%)	77.1 ^b^ ± 2.8	79.0 ^c^ ± 3.5	78.2 ^ab^ ± 2.2	75.2 ^a^ ± 1.8	0.061	0.231	<0.0001
Shin	Total (kg)	0.7 ± 0.07 (1)	0.7 ± 0.08 (1)	0.7 ±0.07	0.7 ± 0.08	0.162	0.220	0.774
	Bone (%)	40.5 ± 2.4	39.5 ± 2.7	41.5 ± 1.8	40.8 ± 2.5	0.047	0.111	0.720
	Muscle (%)	58.4 ± 2.6	59.1 ± 2.8	58.1 ± 1.8	58.2 ± 2.7	0.315	0.507	0.602

^a,b^ Means within the same row with different letters differ (*p* < 0.05), and ^x,y^ means differed with *p* ≤ 0.10 and was considered to be a trend, both reflecting the breed x sex level; Significant *p*-values are presented in bold; BG—Boer Goat; IVG—large frame Indigenous Veld Goat.

**Table 3:** Least-square means and standard error (SE) for the chemical composition of the loins of Boer and large frame Indigenous Veld buck and wether goats.

	Breed						
Proximate Analyses (%)	BG		IVG		Significance (*p*-Values)		
	Bucks	Wethers	Bucks	Wethers	Breed	Sex	Breed × Sex
Moisture	76.3 ± 1.8	75.1 ± 3.0.	76.8 ± 1.7	75.6 ± 3.4	0.099	<0.001	0.350
Protein	20.0 ± 1.79	20.3 ± 2.3	19.6 ± 1.8	20.1 ± 2.5	0.200	0.039	0.855
Fat *	2.2 ± 1.8	2.8 ± 1.7	1.6 ± 1.2	2.7 ± 1.1	0.032	0.001	0.473
Ash	0.9 ± 0.3	1.0 ± 0.2	1.0 ± 1.0.2	1.1 ± 0.2	0.001	0.001	0.140

* Fat % = chemically determined intramuscular fat (IMF); Significant *p*-values are presented in bold; BG—Boer Goat; IVG—large frame Indigenous Veld Goat.
